# System for Estimation of Human Anthropometric Parameters Based on Data from Kinect v2 Depth Camera

**DOI:** 10.3390/s23073459

**Published:** 2023-03-25

**Authors:** Tomasz Krzeszowski, Bartosz Dziadek, Cíntia França, Francisco Martins, Élvio Rúbio Gouveia, Krzysztof Przednowek

**Affiliations:** 1 Faculty of Electrical and Computer Engineering, Rzeszów University of Technology, 35-959 Rzeszów, Poland; 2 Institute of Physical Culture Sciences, Medical College of Rzeszów University, 35-959 Rzeszów, Poland; 3 Department of Physical Education and Sport, University of Madeira, 9020-105 Funchal, Portugal; 4 LARSYS, Interactive Technologies Institute, 9020-105 Funchal, Portugal

**Keywords:** 3D human model, 3D model, human anthropometric parameters, Kinect v2, depth sensor

## Abstract

Anthropometric measurements of the human body are an important problem that affects many aspects of human life. However, anthropometric measurement often requires the application of an appropriate measurement procedure and the use of specialized, sometimes expensive measurement tools. Sometimes the measurement procedure is complicated, time-consuming, and requires properly trained personnel. This study aimed to develop a system for estimating human anthropometric parameters based on a three-dimensional scan of the complete body made with an inexpensive depth camera in the form of the Kinect v2 sensor. The research included 129 men aged 18 to 28. The developed system consists of a rotating platform, a depth sensor (Kinect v2), and a PC computer that was used to record 3D data, and to estimate individual anthropometric parameters. Experimental studies have shown that the precision of the proposed system for a significant part of the parameters is satisfactory. The largest error was found in the waist circumference parameter. The results obtained confirm that this method can be used in anthropometric measurements.

## 1. Introduction

Anthropometric measurements of the human body are applicable to many aspects of human life [1]. Anthropometry is used in scientific research, clinical examinations, and medicine [2,3,4], in dietetics [5], biomechanics [6,7,8], and in the clothing industry [9]. The basis of anthropometry is an anthropometric measurement that requires the application of an appropriate measurement procedure and the use of specialized, sometimes expensive, measuring tools (e.g., anthropometer, measuring tapes, and caliper). In addition, the measurement process is usually complicated, uncomfortable, time-consuming, and requires properly trained personnel [1,2,10,11].

In view of the above, it became reasonable to look for other measurement methods that could support or partially replace the existing techniques or tools. An alternative to classical anthropometry became imaging methods commonly used in medicine (DXA, CT, MRI) [1], as well as estimation of anthropometric parameters using computer vision [11] and a 3D laser body scanner [12]. According to Jaeschke et al. [2], in order to improve the measurement of human body parameters (length, circumference of the trunk, hips, or other body parts), scanners visualizing a three-dimensional human model may prove useful. Liu et al. [13] stated that 3D scanners have fundamentally changed the approach to this type of anthropometric measurement in recent years. In [14], a synthetic data set of human body shapes was used to develop a method for estimating anthropometric parameters using deep learning and neural networks.

In the literature, there can be found some studies in which images from digital cameras [11,15,16], Kinect sensor [17,18,19,20], MoCap systems [21], or professional 3D scanners [2,4] were applied to estimate the individual types of human anthropometric parameters. Most of the mentioned solutions are complex optical systems consisting of multiple cameras, emitters of structured light, or laser beams [2,4,16,21]. They are characterized among others by the ability to perform a relatively quick and three-dimensional scan of the object, high resolution, and high accuracy. Unfortunately, they are expensive to purchase, their application is sometimes complicated, and due to their architecture, they are mainly used in laboratory conditions [22,23].

In the group of systems and tools used to estimate the anthropometric parameters of the human body, the Kinect sensor has found a wide application. The device, characterized by a low price, and equipped with an RGB camera, an infrared camera, and an infrared emitter, has become a significant element of scientific research worldwide [6,24,25].

Among the publications describing the varied application of the Kinect sensor, there are studies in which the authors characterized the sensor in terms of hardware [6], software, as well as procedures for calibration and synthesis of cameras, or methods aimed at improving and smoothing the image obtained by means of the sensor [24,26,27]. Cai et al. [25] described examples in which the device was used in various support systems for industry, detection, recognition and tracking of objects, tracking people and analyzing human activity. The Kinect sensor has also found applications in medicine [28,29,30,31], sport [32,33,34,35,36,37,38], and biomechanics [6], where IMU and EMG sensors have recently become popular methods for improving the accuracy of human movement pattern recognition [39].

It was also used as a tool supporting the process of estimating the anthropometric parameters of the human body. Some studies concerning this issue described the results obtained in systems based on one [9,17,20,40,41], three [42], four [43], and even 16 Kinect sensors [19]. Taking into account the solution with a single depth camera, the estimation of elementary human anthropometric measures was usually made based on the analysis of several images recorded by the sensor [17,41], as well as a three-dimensional model obtained from a partial or complete scan of the human body [9,20].

He et al. [20] developed a system performing anthropometric measurements based on a 3D model of the human body obtained from images captured by the Kinect sensor. The developed system has made it possible to measure the volume of the human body and the circumference of the chest, waist, and hips. The obtained results were compared with the results achieved by other methods for the construction of 3D human scans. The complete body scan was also performed by Kudzia [44], who applied a point cloud to calculate the volume and mass of individual body parts. However, the proposed method has a number of limitations, the most serious being the need for manual segmentation of the human body and the long time needed to perform this procedure. In another study in this field, the results of the application of the Kinect sensor to perform a 3D scan and anthropometric measurements of people wearing clothes and in various body positions were presented [9]. Naufal et al. [41] used in turn the Kinect sensor to calculate the height and surface area of the human body for weight estimation. In the estimation process, linear regression and polynomial regression were applied.

The review of the literature shows that the application of depth sensors as a tool supporting anthropometric measurements of the human body may be justified, but there is a lack of comprehensive solutions that enable the measurement of many anthropometric parameters and are thoroughly tested on many objects. This study aimed to develop a system for estimating human anthropometric parameters based on a three-dimensional scan of the complete body made with an inexpensive depth camera in the form of the Kinect v2 sensor. The developed system builds a 3D human model based on the data obtained from the depth sensor, then performs the segmentation of this model and estimates seven anthropometric parameters featuring the human build. It should be noted that the Kinect v2 Sensor is used only to acquire depth data and can be replaced with another sensor (e.g., Intel RealSense D455, Azure Kinect) that makes it possible to obtain this type of data. Summarizing, the main contributions of this paper can be stated as:

- To develop a system for the estimation of human anthropometric parameters based on the data from a depth camera;
- To develop a method for estimating anthropometric parameters from 3D scans;
- Using and verifying the possibility to estimate anthropometric parameters by the Kinect v2 sensor.

## 2. Materials and Methods

### 2.1. Data Collection

The research included 129 men aged 18 to 28. The men featured a weight at a level of 79.4 ± 11.7 kg and a body height of 180.2 ± 6.5 cm. All participants of the research gave their written consent to the anthropometric examination and consent to perform the 3D body scan.

**Figure 1:**
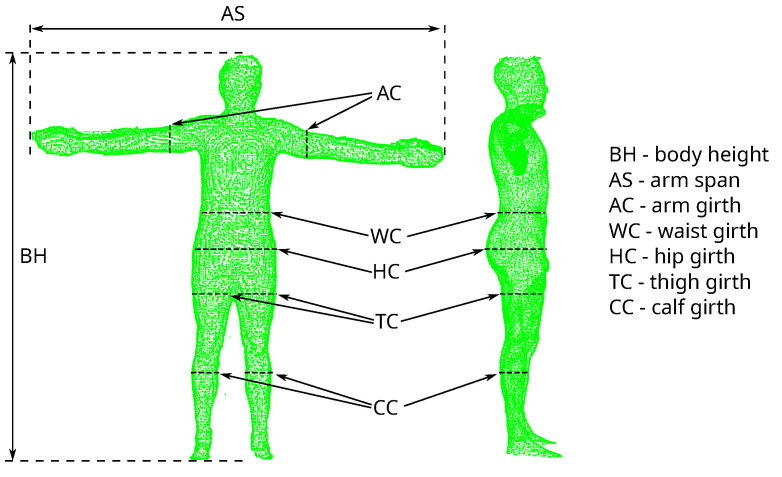
A 3D scan with marked places of estimation of parameters.

All anthropometric parameters were measured according to the International Standards for Anthropometric Assessment (ISAK) procedures [45] and included direct measurement of seven anthropometric parameters (see Figure 1):

- Body height (BH)—the body height was measured with a stadiometer (SECA 213 Hamburg, Germany) with an accuracy of up to 1 mm.
- Arm span (AS)—the subject stood with his back to the wall so that his back, buttocks, and heels touched the wall. The subject then raised both hands horizontally and the fingers of both hands were straightened. Then the left hand with straight fingers touched the corner of the room. The arm span was measured with tape from the corner of the room to a mark on the wall that corresponded to the end of the right hand.
- Waist girth (WC)—measurements of the waist circumference were carried out with anthropometric tape, an approximate midpoint between the lower margin of the last palpable rib and the top of the iliac crest.
- Hip girth (HC)—the hip circumference measured around the widest portion of the buttocks.
- Arm girth (AC)—the subject was in a relaxed standing position with the arms hanging by the sides. The girth of the arm is measured by the anthropometric tape positioned perpendicular to the long axis of the arm at the level of the midpoint between the corner of the acromion and the proximal radial head. The tape should be positioned perpendicular to the long axis of the arm.
- Thigh girth (TC)—the subject stands with his legs slightly apart and his body weight evenly distributed on both feet. The measurement was carried out using anthropometric tape in mid-thigh in a perpendicular plane to the long axis of the thigh so that the flexible tape does not indent the skin excessively.
- Calf girth (CC)—the subject stood with feet slightly apart and body weight evenly distributed. Measurement was made in place of the maximum circumference of the calf in the plane perpendicular to the vertical axis of the leg. The measuring tape has been wrapped so that it does not indent the skin excessively.

### 2.2. System for Estimation of Human Somatic Parameters

**Figure 2:**
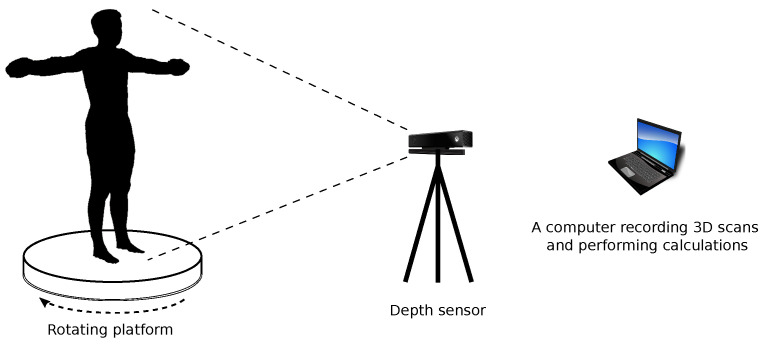
System for scanning human anthropometric parameters.

The system for estimating human somatic parameters (Figure 2) consists of a rotating platform on which the measured person stands, a depth sensor that allows recording a 3D scan, and a PC computer that is used to record 3D data, as well as carrying out calculations related to the estimation of individual parameters. In the proposed solution, Kinect v2 was used as the depth sensor. The 3D scan of the measured person is recorded using a rotating platform (the platform rotates by $360^{\circ }$ with a constant speed), which allows a full 3D scan of the human body. The scanned person should stance in a T-pose and his clothing should be limited to a minimum (e.g., tight-fitting underwear). During scanning the sensor records multiple 3D scans that present the human body from different sides. These scans are analyzed to find correspondence and merge into one 3D scan. This operation is performed on the basis of methods known from the literature and available in point cloud processing libraries [46].

### 2.3. Segmentation of a 3D Scan of the Human Body

**Figure 3:**
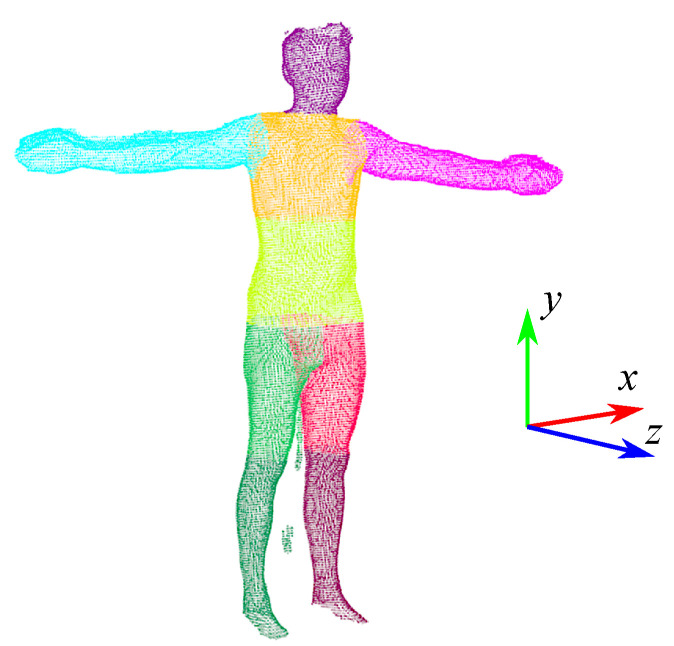
Segmented 3D scan of the human body.

**Figure 4:**
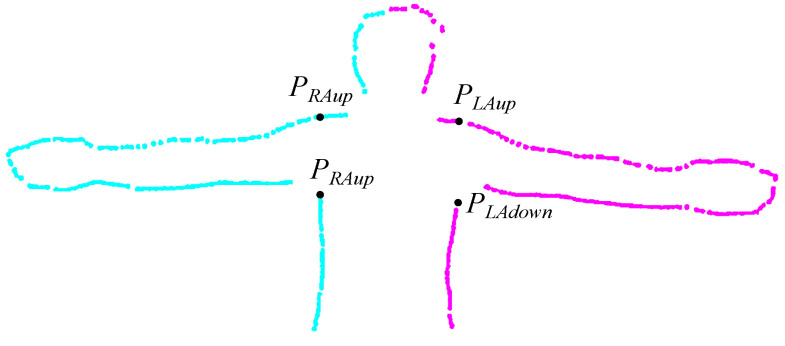
The contour points of the upper body determined using the Concave Hull method projected onto the XY plane.

In order to determine somatic parameters from a 3D scan (point cloud), it is necessary to perform segmentation in order to separate individual body segments. The 3D scan of the human figure is divided into 9 parts (Figure 3): head, upper torso, lower torso, right arm, left arm, right thigh, left thigh, right lower leg, and left lower leg. Segmentation is based on the proportions of individual parts of the body and finding the characteristic features of the human figure. The necessary aspect ratios for individual parts of the body were determined on the basis of measurements carried out on the test group. In the segmentation process, the location of the scan in the coordinate system is important (see Figure 3). To perform the calculations correctly, the scan should be positioned so that the z-axis corresponds to the sagittal axis of the human body, the y-axis corresponds to the vertical axis, and the x-axis corresponds to the transversal axis. First, the geometric center of the point cloud is calculated, which determines the approximate position of the scanned character’s hips. Then the point cloud is filtered so that only points belonging to the hips remain. Among these points, the point ($P_{H}$) with the smallest value of the *z* coordinate is searched. The *y* coordinate of the $P_{H}$ point corresponds to the height for which the hip circumference (HC) is calculated. This height also defines the dividing line for the lower and upper body parts so that the scan can be divided into two parts corresponding to the humans’ upper and lower body. Then, for the points whose value for the *y*-axis is in the range ($P_{H}.y-th_{H},\;P_{H}.y+th_{H}$), the algorithm determines points with the smallest ($P_{H\;minx}$) and largest ($P_{H\;maxx}$) value of the *x* coordinate. The *x* coordinates of these points allow us to calculate $x_{H}=\left(P_{H\;minx}.x+P_{H\;minx}.x\right)/2$, and then determine the points that belong to the right (points with the value of the coordinate *x* less than $x_{H}$) and left (points with the coordinate value *x* greater than $x_{H}$) side of the scanned human. Then, based on the proportions of the body, for the right lower part of the body (right leg) and the left lower part of the body (left leg), the coordinates *y* of the scanned character’s knees are determined, which allows for determining the points belonging to the right thigh, right lower leg, left thigh, and left lower leg. In order to isolate the arms, the points corresponding to the upper body are projected onto the XY plane and processed by the Concave Hull method to determine the points that define the outline of the 2D projection of the upper body (see Figure 4). Then, the contour points obtained in this way are filtered to isolate the points belonging to the right (points with the coordinate value *x* less than $x_{H}$) and the left (points with the coordinate value *x* greater than $x_{H}$) part of the human figure. In the next step, an analysis is performed to determine the points defining the beginning of the right ($P_{RA\;down}$ and $P_{RA\;up}$ in Figure 4) and left ($P_{LA\;down}$ and $P_{LA\;up}$ in Figure 4) of the arm. First, points $P_{RA\;down}$ and $P_{LA\;down}$ are determined, which define the lower beginning of the arms. These points are determined on the basis of the analysis of the directions of normalized vectors, the beginning, and end of which are determined by successive contour points. $P_{RA\;down}$ is defined as the origin of the first normalized vector for which the *x* coordinate is greater than the $th_{dir}$ parameter, with the assumption that the analysis proceeds from the lowest points. Having $P_{RA\;down}$, subsequent points are analyzed in order to find a point ($P_{RA\;up}$) whose coordinate *x* is close to the *x* coordinate of the point $P_{RA\;down}$. Determination of $P_{LA\;down}$ and $P_{LA\;up}$ is performed in a similar way, except that the points belonging to the outline of the left side of the human are analyzed. With the points $P_{RA\;down}$, $P_{RA\;up}$, $P_{LA\;down}$ and $P_{LA\;up}$ the points belonging to the torso, right arm, left arm, and head can be determined. The torso is then split in half to make an upper and lower torso (Figure 3). The calculations were carried out using the PCL library [46].

### 2.4. Estimation of Human Somatic Parameters

With a segmented 3D scan, somatic features can be determined. The height (H) of the human figure is calculated on the basis of the coordinates of the points with the maximum and minimum value for the *y* axis, while the arm span (AS) is calculated on the basis of the coordinates of the points with the maximum and minimum value for the *x* axis. The procedure for determining the remaining somatic features from the 3D scan is as follows:

1. In order to calculate given perimeters, fragments of point clouds are separated from individual segments. These points are determined as follows:
  (a) Arm girth (AC)—the place (point $P_{AC}$) where the circumference is calculated is halfway between the beginning of the arm (defined by points $P_{RA\;down}$ and $P_{RA\;up}$—right arm, $P_{LA\;down}$ and $P_{LA\;up}$—left arm) and elbow (approximate position of the elbow is calculated on the basis of the proportion of the length of the arm to the forearm; this proportion was determined on the basis of the measurements of the test group). Then the points of the arm whose coordinate *x* is in the range $\left(P_{AC}.x-th_{cut},\;P_{AC}.x+th_{cut}\right)$ are projected onto the YZ plane.
  (b) Waist girth (WC)—the place (point $P_{WC}$) where the waist circumference is calculated is estimated based on the measurements of the test group, during which measured the distances between the beginning of the torso (place of hip circumference measurement) and the waist and between waist and the end of the body (beginning of the neck). Torso points whose coordinate *y* is in the range $\left(P_{WC}.y-th_{cut},\;P_{WC}.y+th_{cut}\right)$ are projected onto the plane XZ.
  (c) Hip girth (HC)—the value of the *y* coordinate corresponding to the location of the hip circumference measurement ($P_{H}.y$) is determined during segmentation. Points for which the *y* coordinate is in the range $\left(P_{H}.y-th_{cut},\;P_{H}.y+th_{cut}\right)$ are projected onto the XZ plane.
  (d) Thigh girth (TC) is calculated for points located in the middle of the thigh segment. The $y_{TC}$ coordinate is derived from the points at the beginning and end of the thigh. Points for which the $y_{TC}$ coordinate is in the range $\left(y_{TC}-th_{cut},\;y_{TC}+th_{cut}\right)$ are projected onto the XZ plane.
  (e) Calf girth (CC)—at the beginning, the approximate place of circumference measurement is determined, for this purpose, based on the measurements of the test group, during which the distances between the knee and the calf girth measurement place and the calf girth measurement place and the foot, the $y_{CC}$ coordinate was determined. Among the filtered points, the point ($P_{CC}$) with the smallest value of the *z* coordinate is searched. The *y* coordinate of the $P_{CC}$ point corresponds to the height for which the calf has the greatest circumference. Points for which the *y* coordinate is in the range $\left(P_{CC}.y-th_{cut},P_{CC}.y+th_{cut}\right)$ are projected onto the XZ plane.
2. Using the Convex Hull method, an ordered list of points is determined from the points projected onto the plane;
3. The perimeter is calculated from the equation:
  (1) $$L=\sum_{k=1}^{n}d\left(P_{k},P_{k+1}\right),$$
  where $P_{n+1}=P_{1}$ and $d(P_{1},P_{2})$ is the Euclidean distance between the points $P_{1}$ and $P_{2}$.

### 2.5. Statistical Analysis

The study used basic statistical measures, i.e., the arithmetic mean, standard deviation, median, and first and third quartiles. In addition, two indices of absolute difference (*d*) and relative difference (Δ) were determined:

(2) $$d=\frac{1}{n}\sum_{i=1}^{n}\left(GS_{i}-DC_{i}\right)$$

(3) $$\Delta =\frac{d}{\frac{1}{n}\sum_{i=1}^{n}\left(GS_{i}\right)},$$

where: *n*—total number of patterns, GS—gold standard value, and DC—estimated value.

Statistical evaluation of the significance of the differences was performed using the U Mann–Whitney test, taking *p* < 0.05 as significant. In addition, for a detailed comparison of the obtained results with the gold standard, analysis was performed using Bland–Altman plots. The coefficient of repeatability (CR), defined as the two standard deviations of the differences of the paired parameters, and the coefficient of variance (CV), expressed in percentage as the quotient of the standard deviation of the differences of the individual paired parameters by the mean, were determined. Additionally, Pearson correlations between measured and estimated values were calculated. Statistical analysis was performed in the GNU R software [47].

## 3. Results and Discussion

The experimental study consisted of verifying the accuracy of estimating selected anthropometric parameters calculated using the presented algorithm. The values of the obtained parameters were compared to direct measurement, which was considered as a gold standard (GS). The estimated results, along with the errors, are presented in Table 1.

The study shows that the most accurately estimated parameter was the body height parameter for which d=0.002 m while Δ=0.1%. It should also be noted that the difference with regard to GS did not show statistical significance. Analyzing the median values, it is noted that the estimated values are close to the GS values. The situation is similar for quartiles Q1 and Q3. Analyzing dispersion, it is noted that an identical standard deviation was achieved for 5 of the 7 parameters. Different standard deviations were noted for arm span and waist girth. The remaining differences show statistical significance. The largest difference was observed for waist circumference d=−0.074 m and Δ=−9.2%. Correlation analysis of the two measurements showed a very strong positive relationship (Table 2). Correlation coefficients ranged from r=0.61 for arm girth to r=0.97 for body height. For four parameters, a full correlation of the measurement results are found (r>0.9).

Analysis using the Bland–Altman method (Table 2) showed that as many as five parameters had a recurrence rate of less than CV<5%. For calf girth, a CV=5.1% was recorded, while the highest for arm girth was CV=8.8%. The best precision is characterized by the estimation of the body height parameter CV=1.1%. The coefficient of repeatability was very small and its value was equal to 0.04 for four parameters (body height, calf girth, hip girth, and thigh girth) and 0.06 for three parameters (arm span, arm girth, and waist girth).

**Figure 5:**
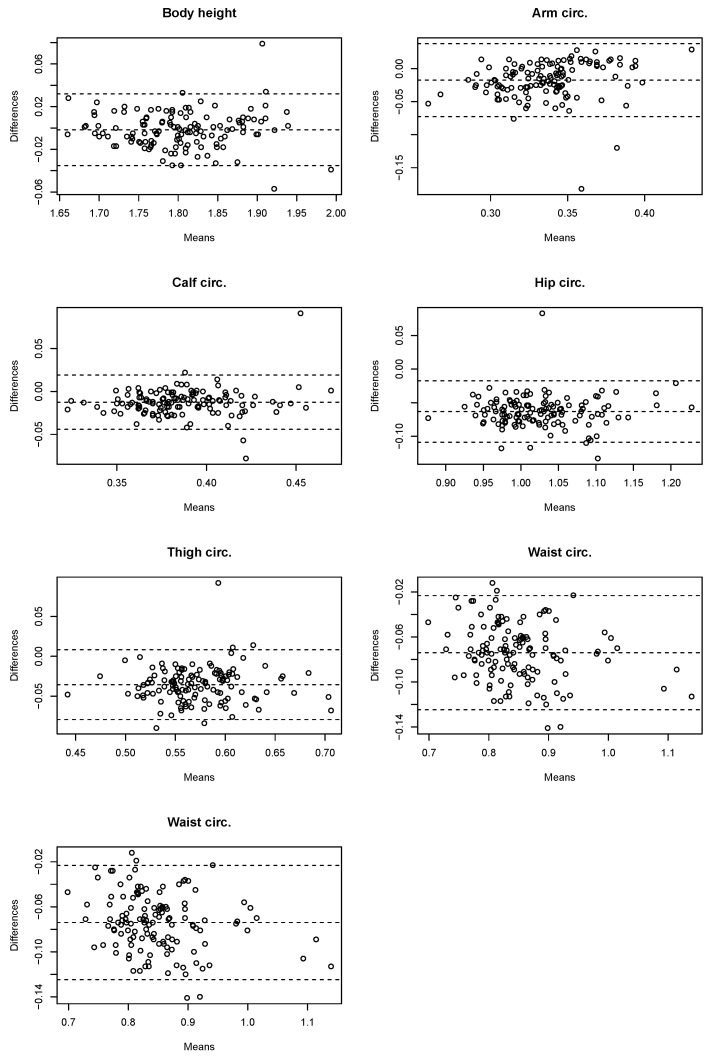
Bland–Altman plots for anthropometric parameters.

In order to visualize in detail the differences between the calculated values from the 3D scan and the measured values (gold standard), analysis using Bland–Altman charts was used (Figure 5). The vast majority of measurements fall within the CV range, with only isolated cases deviating from the gold standard.

The comparative analysis carried out showed that the estimation of selected anthropometric parameters using the depth sensor generates acceptable errors. The main quality criterion adopted in the presented solution was the comparison with direct measurements of anthropometric parameters. Comparison of the obtained errors with errors generated by models presented by other researchers is not obvious due to the use of different criteria for evaluating methods. An interesting study was presented by Kahelin et al. (2020) [8], where they also used a 3D scan model from the reconstruction of 2.5D information capture from Kinect. The errors they obtained for hip girth (0.019 m) and thigh girth (0.013 m) were smaller than the errors presented in this paper: hip girth (0.063 m) and thigh girth (0.036 m) (Table 1).

Another paper that allows a direct comparison with the proposed method is that of [2], which presents a laser-based body surface scanner (Virtual smart XXL). This scanner was also compared to manual measurements and the differences and correlation were evaluated. The results for men were also more accurate than the results presented in this paper. The correlation coefficients of the estimated measurements with the manual measurements were 0.97 for waist girth and 0.97 for hip girth, whereas, for the method presented in this paper, the correlation results were 0.94 for waist girth and 0.93 for hip girth (Table 2). However, it should be noted that the laser scanner used in [2] is a more accurate measurement device relative to the Kinect camera-based scanner (Table 1).

A paper that also used a Kinect camera as a tool to determine a 3D scan of the human silhouette was by Tong et al. 2012 [42]. Tong and co-authors used two Kinects to capture the upper part and the lower part of a human body, respectively, and a third Kinect, placed on the opposite side, is used to capture the middle part of the human body. It is worth noting that the scanned subjects were wearing clothes. The results obtained for waist girth (0.062 m) and for hip girth (0.038 m), were also smaller than the errors presented in this work (Table 1).

The Kinect sensor is very often used as a tool to scan either the entire figure or selected body segments [22,28,29,40,41,43]. However, the direct comparison of errors is complicated by the use of different quality criteria. For example, in the work [22], the TEM criterion (the relative technical error of measurement) was defined, whose value was calculated at 0.88%. In the work of [28], where a 3D foot scanner based on a Kinect camera was presented, the RMSE (Root mean squared error) criterion was used. The RMSE error was calculated against a high-resolution laser scanner and was 2.8 mm. In another paper [40], where Kinect v2 was also used to estimate selected anthropometric parameters, the authors inferred that the differences between the estimated values and the traditional measurements show statistical significance, which confirms the results we obtained, presented in Table 1. Naufal et al. [41] studied a total of 147 subjects. The results measured manually were compared with the automatic estimation realized with the Kinect camera. The difference between the estimation and the manual measurement ranked at 1.04% and showed statistical significance.

## 4. Conclusions

This paper presents and tests a method for measuring selected anthropometric parameters using an inexpensive depth camera in the form of the Kinect v2 sensor. In order to evaluate the method, a statistical analysis was carried out in the form of a U Mann–Whitney test and Bland–Altman charts. Experimental studies have shown that the accuracy of the proposed system for a significant part of the parameters is satisfactory (Δ<7%). The largest error was in the waist circumference parameter. The results obtained confirm that the method can find application in anthropometric measurements. The use of newer devices, such as Azure Kinect, should allow for more accurate parameter estimates.

Limitations of the work are related to the validation of the method. The proposed method is tested only for selected anthropometric parameters. Another limitation is the research group. Parameter estimation was performed for the male gender. The method was not tested in a group of women. The research also did not take into account non-standard cases, such as body deformities, missing or shorter limbs, etc. In such cases, the system may give incorrect results.

Future work will be related to the development of new functionalities of the algorithm and the use of machine learning methods to classify body composition components and somatotype components. It is also planned to test the proposed method with other depth sensors, e.g., Azure Kinect. In addition to using a more accurate sensor, we will also work on improving the accuracy of the method itself, for this purpose various filtration and smoothing algorithms will be tested. Another important element of future work will be the inclusion of the female gender in the study.

## 5. Patents

T. Krzeszowski, K. Przednowek: “Method for estimating somatic features, somatic indicators, somatotype components, somatotype and body composition components with the use of depth sensor”, Polish patent publication PL240075B1, 2022.

## Figures and Tables

**Table 1:** Characteristics of the anthropometric parameters for GS and DC methods (*N* = 129).

Parameter	GS—Gold Standard					DC—Depth Camera Estimation					d	Δ	*p*
	$\bar{x}$	sd	Me	Q1	Q3	$\bar{x}$	sd	Me	Q1	Q3			
arm span (m)	1.84	0.07	1.84	1.80	1.89	1.83	0.08	1.83	1.78	1.89	−0.012	−0.7%	0.001 *
body height (m)	1.80	0.07	1.80	1.76	1.85	1.80	0.07	1.80	1.76	1.84	−0.002	−0.1%	0.224
arm girth (m)	0.35	0.03	0.34	0.33	0.36	0.33	0.03	0.33	0.31	0.35	−0.017	−5.9%	0.001 *
calf girth (m)	0.39	0.03	0.39	0.38	0.41	0.38	0.03	0.38	0.36	0.40	−0.013	−3.4%	0.001 *
hip girth (m)	1.06	0.06	1.05	1.02	1.09	0.99	0.06	0.98	0.95	1.02	−0.063	−6.4%	0.001 *
thigh girth (m)	0.59	0.04	0.59	0.56	0.61	0.55	0.04	0.55	0.53	0.58	−0.036	−6.6%	0.001 *
waist girth (m)	0.89	0.08	0.88	0.84	0.92	0.81	0.07	0.80	0.77	0.84	−0.074	−9.2%	0.001 *

$\bar{x}$—mean; *sd*—standard deviation; Me—median; Q1—first quartile; Q3—third quartile; d—index of absolute difference; Δ—index of relative difference %; *p*—statistical probability; *—statistical significance.

**Table 2:** Results of correlation and Bland–Altman analysis.

Parameter	${\bar{x}}_{\mathrm{GS}+\mathrm{DC}}$	*r*	${\mathrm{sd}}_{\mathrm{GS}-\mathrm{DC}}$	CR	CV
arm span (m)	1.84	0.94	0.03	0.06	1.6%
body height (m)	1.8	0.97	0.02	0.04	1.1%
arm girth (m)	0.34	0.61	0.03	0.06	8.8%
calf girth (m)	0.39	0.84	0.02	0.04	5.1%
hip girth (m)	1.03	0.93	0.02	0.04	1.9%
thigh girth (m)	0.57	0.87	0.02	0.04	3.5%
waist girth (m)	0.85	0.94	0.03	0.06	3.5%

${\bar{x}}_{GS+DC}$—mean of GS and DC values; *r*—correlation coefficient; ${sd}_{GS-DC}$—standard deviation of differences between GS and DC values; CR—coefficient of repeatability; CV—coefficient of variance.

## Data Availability

The data presented in this study are available upon request from the corresponding author.
